# Degradation Kinetics and Mechanism of Lithospermic Acid under Low Oxygen Condition Using Quantitative ^1^H NMR with HPLC-MS

**DOI:** 10.1371/journal.pone.0164421

**Published:** 2016-10-24

**Authors:** Jianyang Pan, Xingchu Gong, Haibin Qu

**Affiliations:** Pharmaceutical Informatics Institute, College of Pharmaceutical Sciences, Zhejiang University, No. 866 Yuhangtang Road, Hangzhou, 310058, China; Weizmann Institute of Science, ISRAEL

## Abstract

A novel quantitative ^1^H NMR (Q-NMR) combined with HPLC-MS method has been proposed for investigating the degradation process of traditional Chinese medicine (TCM) components. Through this method, in-situ monitoring of dynamics degradation process of lithospermic acid (LA), one of the popular polyphenolic acids in TCM, was realized under low oxygen condition. Additionally, this methodology was proved to be simple, rapid and specific. Degradation kinetic runs have been carried out to systematically investigate the effects of two key environmental factors, initial pH values and temperatures. Eight main degradation products of LA were detected, seven of which were tentatively structural elucidated with the help of both NMR and LC-MS in this work and salvianolic acid A (Sal A) was the primary degradation product of LA. A possible degradation pathway of LA was proposed, subsequently. The results showed that the degradation of LA followed pseudo-first-order kinetics. The apparent degradation kinetic constants increased as the initial pH value of the phosphate buffer increased. Under the given conditions, the rate constants of overall degradation as a function of temperature obeyed the Arrhenius equation. Our results proved that the Q-NMR combined with HPLC-MS method can be one of the most promising techniques for investigating degradation process of active components in TCM.

## Introduction

The chemical complexity and vagueness of effective compounds are major impediments to the development of traditional Chinese medicine (TCM) into modern medicine in comply with pharmaceutical standards accepted worldwide. Multi-components and multi-species of many TCMs are accountable for most of these challenges, additionally, the deficiencies in modern standardization and quality control methods for TCM are also important contributing factors [1]. In most cases, the TCM production process consists of the following indispensable units: extraction, concentration, purification, drying and preparation. Heating and acid/alkali adjustment are commonly used in extraction step, which may cause chemical reactions such as degradation of active components, this will directly affect the quality of TCM [2], thus its clinical safety and efficacy cannot be guaranteed. Therefore, the study on degradation process of active components in TCM is vital for improving quality control and quality consistency of TCM during manufacture and resulted pharmaceutical product.

Mechanism and kinetics are two major questions to be addressed in a degradation study. As one of the most powerful analytical tools in study of degradation mechanism, HPLC-MS is known for rapid compound identification with the help of widely used chemical databases [3–5]. In order to perform kinetic modeling in degradation study, both reactant and degradation products should be quantitatively analyzed and HPLC-UV is the dominant technique used [6–8]. Despite of all the superb characteristics of HPLC methods, the requirement of reference compounds is a limitation of its application in quantitative analysis. Without reference compounds, chromatographic results are reported on area percent rather than weight percent, which are not accurate enough for certain studies. Meanwhile, the preparation of reference compounds for degradation products is often time consuming and sometimes impossible when these compounds are unstable. Furthermore, these analytes may undergo chemical conversions when exposed to air during sample preparation in HPLC analysis. Spectroscopic coupled with multivariate curve resolution (MCR) methods had also been applied in degradation studies [9, 10]. Nevertheless, spectra and concentration matrix of degradation products resolved from MCR often belonged to mixture of compounds rather than a single compound. Furthermore, if the reactant and degradation products show similar spectroscopic property, these methods would fail to resolve. Another frequently overlooked question is that most researchers ignored the effect of oxygen on degradation process. Their reaction system is always exposed to the air, while the operation environment in real industrial manufacturing process of TCM is under low oxygen condition since it is relatively sealed. Therefore, the kinetic results obtained from their laboratory studies offered little instructive information to real industrial manufacturing and quality control of corresponding TCM. As a consequence, there is an urgent need in developing simple, rapid and in-situ research techniques for the study of degradation process of active components in TCM.

One of the major advantages of quantitative NMR (Q-NMR) spectroscopy is its primary analytical characteristics [11]. It is in-situ, avoiding analytes conversions during sample preparation in HPLC analysis. NMR tube is deep in length and has quite small cross-sectional area, so it can be treated as a small reactor with less contact to air. Meanwhile, NMR is structurally rich and non-intrusive, which makes the technique extremely useful to identify and characterize reaction products, gain insights into the reaction mechanisms and determine reaction kinetics. Several studies have reported on reaction monitoring using NMR spectroscopy [12–16]. In recent years, Q-NMR combined with chemometrics methods have been successfully applied in analysis of herbs and herbal preparations [17–19]. We have previously established a Q-NMR method for hydrolytic kinetic investigation of Salvianolic acid B (Sal B) [20]. Q-NMR methods have been demonstrated to be simple, rapid, efficient and reliable.

**Fig 1:**
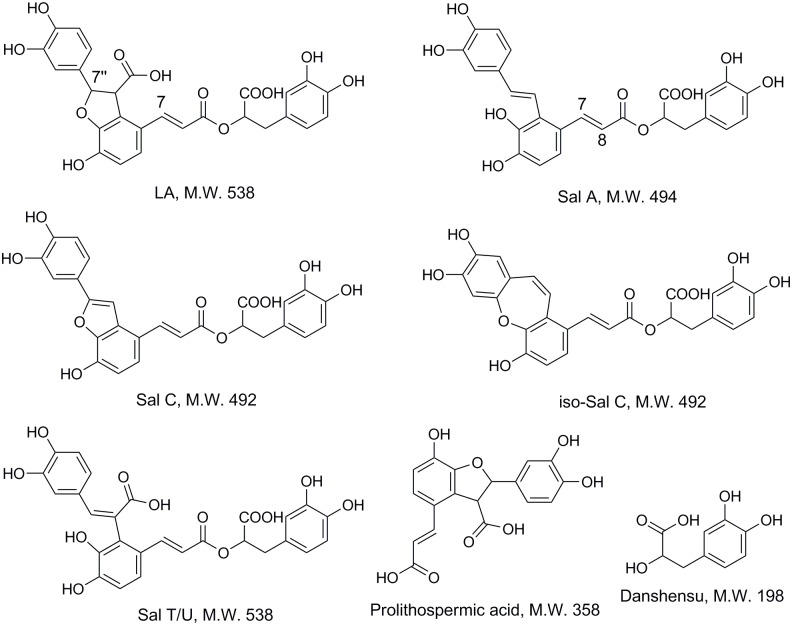
Structure of LA and some of its degradation products.

In this work, Q-NMR combined with HPLC-MS method was proposed to investigate the degradation process of Lithospermic acid (LA, Fig 1) as an example. LA is one of the popular polyphenolic acids existed in several medicinal plants, such as *Tournefortia sarmentosa Lam*. [21], *Salvia miltiorrhiza Bge*. (Danshen) and *Origanum vulgare L*. [22]. It is considered to be the major active constituent of *T*. *sarmentosa*, with its content being about 0.6% w/w in the dried herb [21]. LA possesses the effects of anti-oxidation, anti-inflammation [23], anti-lipid-peroxidative [21] and anti-neuro-inflammationm [24]. Several researchers have illustrated that Salvianolic acid A (Sal A) is degradation product of LA [25, 26]. In recent years, there is a growing interests in Sal A due to its potent bioactivities. It is reported to show highest antioxidant activity among the salvianolic acids in Danshen [27], and possess protective effects in cardiac diseases [28, 29]. The content of Sal A in Danshen is very low. Several methods have been reported to prepare Sal A through the degradation of Sal B [30–34]. Wang et al. [25] found out that compared to Sal B, LA could more easily convert to Sal A and generate fewer other products under the same conditions. Up to now, none reports have been found on the degradation kinetics of LA, not to mention the kinetics of LA transform to Sal A. A possible reason was that Sal A was not stable in aqueous solutions and could be easily oxidized [4].

In current study, a novel Q-NMR combined with HPLC-MS method was established to investigate the degradation process of LA in low oxygen condition. NMR tube was used as reactor and argon was bubbled into the bottom of the NMR tube to vent oxygen. The Q-NMR method was used to monitor the concentration of LA and Sal A in process samples of degradation reaction, which was in situ, without sampling. NMR along with HPLC-MS method was used for structure elucidation of degradation products. A possible degradation pathway of LA was proposed. Furthermore, the concentration of LA and Sal A were fitted and the degradation rate constants were obtained.

## Materials and Methods

### Reagents and chemicals

LA (lot. 140107, 99.05%), Sal A (lot. 140710, 99.53%), Danshensu (lot. 141024, 99.10%), Salvianolic acid C (Sal C, lot. 130902, 99.53%) were purchased from Shanghai Winherb Medical S & T Development Co., Ltd. (Shanghai, China). The mixture of Salvianolic acid T and U (Sal T/U) was kindly provided by Tasly Pharmaceutical Co., Ltd. (Tianjin, China). Deuterium oxide, 99.9 atom % D (D_2_O), contains 0.05 wt. % 3-(trimethylsilyl)propionic-2,2,3,3-d_4_ acid sodium salt (TSP), Phosphoric acid, Sodium phosphate dibasic dehydrate and Sodium phosphate monobasic dehydrate were bought from sigma-Aldrich Corporate (St. Louis, MO, USA). HPLC grade formic acid was purchased from ROE Scientific Inc. (Newark, DE, USA). HPLC grade acetonitrile and methanol were obtained from Merck KGaA (Darmstadt, Germany). NMR tube (Norell 502–7) and rubber tube cap (Norell SEPTA-5-W) were purchased from Norell, Inc. (Landisville, NJ, USA). Lumbar puncture needle (0.7 mm×170 mm) was purchased from Shanghai SA Medical & Plastic Instruments Co., Ltd. (Shanghai, PR China). High purity argon was purchased from Shanghai Wugang gas Co., Ltd. (Shanghai, PR China). Ultrahigh-purity water was produced using a Millipore Milli-Q System (Milford, MA, USA).

### Experimental procedure

**Fig 2:**
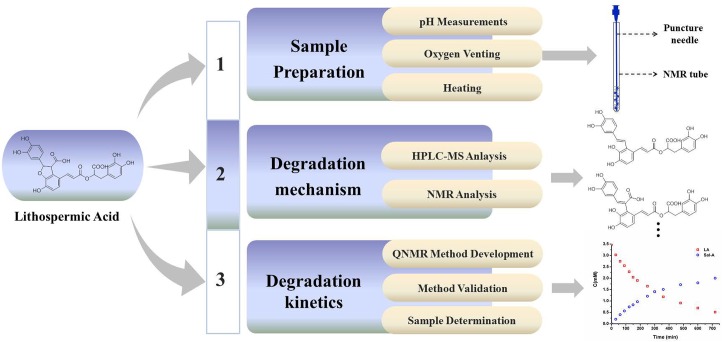
Schematic representation of experimental procedure.

Overall experimental procedures were presented in Fig 2. It consisted of three sections as follows: (1) sample preparation, (2) structure elucidation of degradation products and proposing degradation pathway of LA, (3) degradation kinetic study of LA and Sal A.

### Sample preparation

LA was accurately weighted, dissolved using a mixture of 90% 200 mM phosphate buffer and 10% D_2_O (containing TSP as the internal standard for chemical shift calibration and quantitative analysis). 500 μL of the test solution was added to the NMR tube, and high purity argon was bubbled into the bottom of test solution through a lumbar puncture needle for 2 minutes to vent oxygen. After that, the NMR tube was rapidly sealed up with a rubber cap.

**Fig 3:**
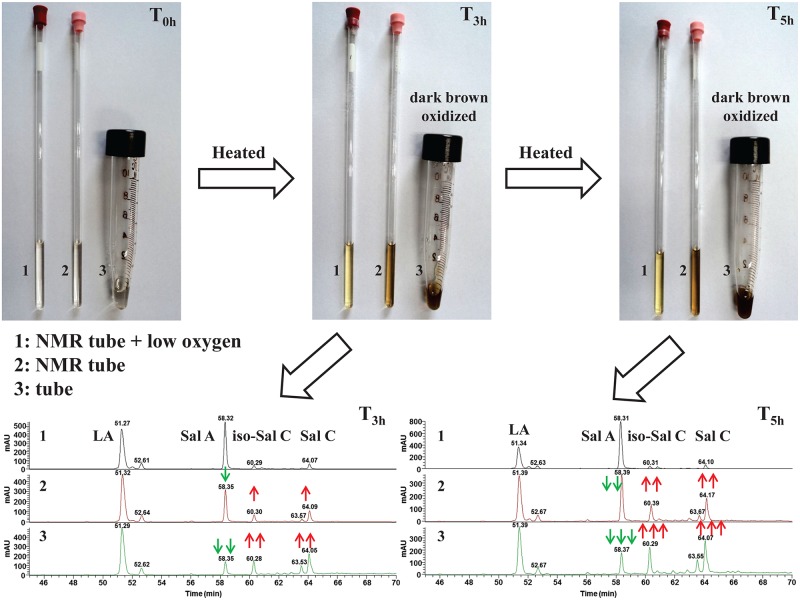
Degraded Samples prepared using different methods.

Degraded Samples prepared using different methods were shown in Fig 3. All samples were heated in a thermostat bath set at 91°C. LA solution was prepared using the method proposed in this work in sample set 1. LA solution was added into NMR tube without venting oxygen in sample set 2. LA solution was added into tube without venting oxygen in sample set 3. The colors of sample solutions in sample set 1 were much lighter than others. According to the results of Q-NMR and LC-MS, a portion of Sal A were oxidized in sample set 2, while the concentration of Sal A were very low in sample set 3.

### HPLC-MS^n^ instrumentation and conditions

HPLC analysis was carried out on an 1100 Series HPLC system (Agilent, Waldbronn, Germany) with diode array detector using an XBridge Shield RP18 column (2.1 mm×150 mm, 3.5 μm, Waters). The temperature of column was maintained at 35°C. UV spectra were recorded from 190 to 400 nm and the detection wavelength was set at 280 nm. The flow rate was 0.2 mL/min, and an in-line filter was used before the analytical column. A gradient elution of mobile phase A (0.1% aqueous formic acid in water) and B (acetonitrile containing 0.1% formic acid) was used. The gradient was as follows: started at 98% A and 2% B, then to 85% A and 15% B at 15 min, 80% A and 20% B at 35 min, 75% A and 25% B at 45 min, 55% A and 45% B at 65 min, 10% A and 90% B at 66 min, kept with 10% A and 90% B from 66 to 70 min. After that, the system was restored to initial conditions in 25 min.

HPLC/MS^n^ analysis was performed with an Agilent 1100 Series HPLC and LCQ Deca XP^plus^ ion trap mass spectrometer (Thermo Finnigan, San Jose, CA, USA) equipped with electrospray ionization (ESI) source, with Xcalibur 1.3 controlling software. Nitrogen (N_2_) was used as the sheath and auxiliary gas, and helium (He) was used as the damping and collision gas. HPLC conditions were the same as described above. The key MS conditions were as follows: negative ion mode, mass range *m/z* 95–1000. The data-dependent MS/MS and MS^n^ events were performed on the most intense ions detected in full scan and MS/MS. ESI parameters were as follows: source voltage, 3 kV; sheath gas, 30 arbitrary units (arb); auxiliary gas, 10 arb; capillary voltage, -15V; capillary temperature, 350°C; tube lens, -30 V.

### NMR instrumentation and conditions

Quantitative NMR analysis was carried out on a Bruker Avance III (500.13M) NMR spectrometer equipped with a 5 mm 1H/D-BBO probehead (Bruker BioSpin GmbH, Rheinstetten, Germany). All the samples were locked individually on 90% H_2_O + 10% D_2_O and were measured at 293.0 K. Gas flow was set at 400 L/h. The spectra were acquired in 32 scans using 32 k data points by noesypr1d pulse program with water signal suppression. The 90° pulse width was set to 14.62 μs and the acquisition time was 2.04 s. The spectra width was set to 8013 Hz. The longitudinal relaxation time T_1_ was determined for the protons of interest (Table 1, Fig 1). Relaxation delay time (D_1_) was optimized for the T_1_ of the longest relaxing TSP nuclei, to ensure maximum recovery of the transverse magnetization, and D_1_ was set to 15 s.

**Table 1:** T_1_ values of monitored protons (tested at 293 K).

Compound	Peaks	Protons	Numbers of protons	T_1_
LA	1–2	H-7”	1	1.63 s
LA	1–1	H-7	1	1.39 s
Sal A	3–2	H-8	1	639 ms
Sal A	3–1	H-7	1	604 ms
TSP			9	3.80 s

### Validation procedures

The stock solution (5.56 mg/mL) was prepared by dissolving an appropriate amount of LA in 200 mM phosphate buffer (pH 5.29). The calibration curve was made using seven standard solutions of different concentrations (5.00, 4.00, 3.00, 2.00, 1.00, 0.500, 0.100 mg/mL). The standard solutions were prepared by diluting an appropriate volume of stock solution with the phosphate buffer and D_2_O (containing TSP as the internal standard for chemical shift calibration and quantitative analysis) was added to volume percentage of 10% for each sample. Each solution was analyzed twice. The peak area values were plotted against the corresponding analyte concentrations to obtain the linear calibration. Intraday precision of the method was determined by measuring six sample of LA (2.00 mg/mL) on the same day.

### Degradation experiments of LA

Twelve or more test tubes were laid in a thermostat bath at predefined temperature and were periodically withdrawn during a kinetic run. Withdrawn samples were rapidly cooled in ice to quench the reaction and were stored in an ice bath until analysis within 2 h. Each study was comprised of twelve or more assays spaced to provide change of ~ 0.1 $C_{1}^{0}$ per samples. After NMR analysis, degradation samples were diluted with an equal volume of 0.3 M phosphoric acid. 2 μL diluted samples were injected into LC-MS^n^ system for tentative structure elucidation of the degradation products.

The influences of temperature and pH values on degradation of LA were investigated. The influence of temperature on degradation was investigated in phosphate buffer solutions at a pH value of 4.75. The reaction rate constants were calculated at 80, 91 and 100°C, respectively. The effect of pH values on degradation was determined at 91°C in phosphate buffer solutions. Specific experimental conditions were listed in Table 2. All the pH measurements were performed on a pH meter (S40 SevenMulti, Mettler-Toledo GmbH, Greifensee, Switzerland) equipped with combination pH electrode (InLab Expert Pro).

**Table 2:** Experimental conditions and kinetic constants for LA degradation.

Exp. No.	C (mg/mL)	T (°C)	pH value	*k*_r1_ (h^-1^)	*r*^2^
I	2.00	80	4.75	0.0436±0.0005	0.998
II	2.00	91	4.75	0.168±0.004	0.994
III	2.00	100	4.75	0.491±0.008	0.996
IV	4.00	91	5.85	0.182±0.003	0.995
V	4.00	91	6.95	0.228±0.002	0.999

### Data Processing

According to the results of preliminary experiment, supposing the degradation of LA followed irreversible first-order reaction kinetics, ordinary differential equation of LA concentration was obtained as follows:

$$\frac{dC_{1}}{dt}=-k_{r1}\cdot C_{1}$$ (1)

where *C*_1_ is the concentration of LA and k_r1_ is the of LA.

Therefore, the concentration of LA could be described by Eqs (2) and (3)

$$C_{1}^{t}=C_{1}^{0}\cdot e^{-k_{r1}\cdot t}$$ (2)

$$\text{ln}\frac{C_{1}^{t}}{C_{1}^{0}}=-k_{r1}\cdot t$$ (3)

where $C_{1}^{0}$ is the initial concentration of LA.

For Sal A, it was degraded from LA and would transform to Sal C and other isomers. Assuming all these reactions follow irreversible first-order reaction kinetics, ordinary differential equation of Sal A was obtained as follows:

$$\frac{dC_{2}}{dt}=k_{r2}\cdot C_{1}-k_{r3}\cdot C_{2}$$ (4)

where *C*_2_ is the concentration of Sal A, k_r2_ is the rate constant of LA transform to Sal A, k_r3_ is the rate constant of Sal A transform to other compounds.

Because the initial concentration of Sal A was 0, Eq (5) can be derived from Eqs (2) and (4):

$$\frac{C_{2}^{t}}{C_{1}^{0}}=\frac{k_{r2}}{k_{r1}-k_{r3}}\cdot \left(e^{-k_{r3}\cdot t}-e^{-k_{r1}\cdot t}\right)$$ (5)

The influence of temperature on reaction rate constant was given by Arrhenius equation:

$$\text{ln }k_{r}=\text{ln }A-\frac{E_{a}}{RT}$$ (6)

where *A* represents frequency factor, *E*_a_ stands for activation energy, *R* is ideal gas constant (8.314 J/mol·K) and *T* is temperature (K).

All data fitting were carried out using OriginLab (Pro 8, OriginLab Corp., Northampton, MA).

## Results and Discussion

### Identification of degradation products

**Fig 4:**
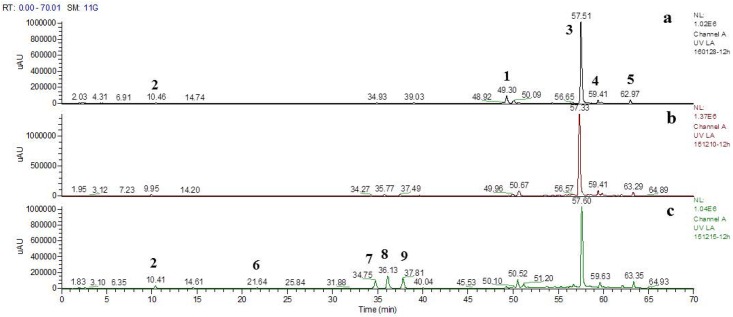
280 nm UV chromatograms of degraded samples obtained under different initial pH values. (a) Exp. No. II; (b) Exp. No. IV; (c) Exp. No. V.

HPLC chromatograms (280 nm) of degraded sample heated 12 h in Exp. No. II, IV and V (for experimental condition details see Table 2) were shown in Fig 4. A tentative assignment of the degradation products was made on basis of quasi-molecular ion [M−H]^−^ and fragment ions obtained from LC—MS^n^ experiments.

The MS of Peak 2 showed [M-H]^-^ at 197 and [2M-H]^-^ at 395, it was identified as danshensu. The MS of Peak 3 showed [M-H]^-^ at 493 and [2M-H]^-^ at 987, it was identified as Sal A. The MS of Peak 5 showed [M-H]^-^ at 491 and [2M-H]^-^ at 983, it was identified as Sal C. In addition, these compounds had been further determined by comparison of retention times with those of reference compounds. MS quasi-molecular ion of peak 4 was the same as peak 5, according to the results in previous work [4], peak 4 was identified as iso-salvianolic acid C (iso-Sal C). The MS of peak 6 showed [M-H]^-^ ion at 357, according to the results in Kan et al.’s [31] and Guo et al.’s [6] studies, peak 6 was identified as prolithospermic acid.

Peak 7, 8 and 9 were three degradation products easily generated at higher initial pH values. They all had the same [M-H]^-^ at m/z 537, which suggested that they were isomers (M.W. 538). The MS/MS and MS^3^ spectra were also similar. They first lost 44u (CO_2_) from the [M−H]^−^ ion to form the abundant [M−H−CO_2_]^−^ ion at m/z 493, and then lost 198u (danshensu) to yield m/z 295, respectively. Compared to retention times with those of reference compounds, peak 8 and 9 were identified to Sal U and Sal T, which was in consistence with the results of Li et al.’s work [35]. MS information could not support the structure elucidation of peak 7 and it was assigned as isomer of LA. Sal U and Sal T were detected as degradation products of LA for the first time in this work.

Sal A was primary degradation product of LA in our experiments. Nevertheless, constitutes of degradation products changed at different experimental conditions. Areas of peak 7–9 increased as the initial pH value of phosphate solution increased. In production of corresponding TCM preparations, process parameters could be adjusted according to production targets. If Sal A is the production targets, solution pH values should be reduced and the reaction system should be kept under low oxygen condition.

### Proposed degradation mechanism

**Fig 5:**
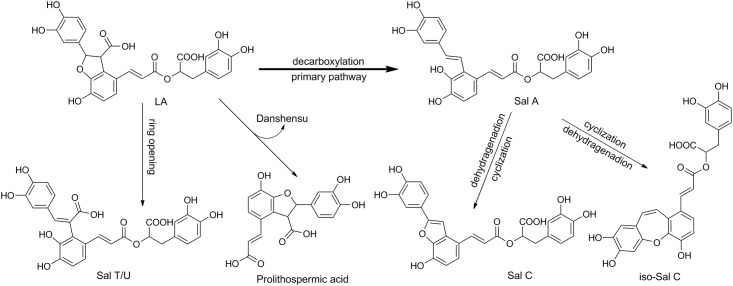
Proposed degradation pathway for LA.

After the identification of the various LA degradation products, a possible pathway of LA degradation in water was proposed, as shown in Fig 5. LA could easily degrade to Sal A through ring opening of benzofuran and decarboxylation. Sal A could further be oxidized to Sal C and its isomers, it was in accordance with previous study [4]. As NMR tube was not sealed, small amount of oxygen might exist in NMR tube, thus, a small part of Sal A was oxidized to Sal C and its isomers. Alternatively, LA could undergo the furan ring-opened to form Sal U and Sal T as the initial of pH of phosphate solution increased. LA could also undergo ester bond hydrolysis to produce danshensu and Prolithospermic acid. Some degradation pathways of LA had been reported by other researchers [25, 31, 32], including decarboxylation and the hydrolysis of the ester bond. While the pathway of ring-open of benzofuran in the parent compound LA to generate Sal T/U was detected for the first time in this work.

### Determination of LA and Sal A using Q-NMR

**Fig 6:**
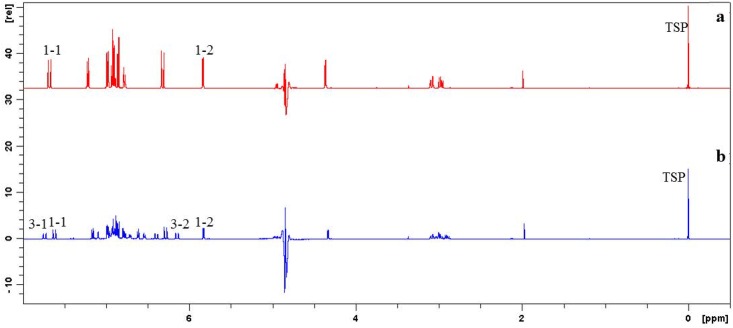
^1^H NMR spectra of LA. (a) and degraded sample heating 5h in Exp. No. II (b).

Typical ^1^H NMR spectra of LA (A) and the degraded samples (B) were shown in Fig 6. Quantitative peaks were selected through comparing ^1^H NMR spectra of standard compounds with those of degraded samples. Two pairs of peaks could be used for quantification of LA (1–1, 1–2) and Sal A (3–1, 3–2). Chemical shift of quantitative peaks upfielded slightly along with the degradation went on. Assignment of quantitative peaks also referred to Zhou et al.’s [26] and Xiao et al.’s works [36]. For ascertaining specificity of the quantitative peaks, 2D NMR (COSY and HSQC) were adopted, supporting the assignment of peaks used for the quantification. LA and Sal A could be quantified using the established Q-NMR method.

### Method validation

The linearity of the Q-NMR method was proved using linear correlation of the peak area values and seven standard solutions of LA in the concentration range of 0.100–5.00 mg/mL (r^2^ = 0.9997 or 0.9994 for peak 1–1 and 1–2). As quantitative NMR is a primary ratio method, linearity is of little significance for assessing the specific detector response factor for different analytes. The experiment was mainly aimed at checking the system stability for the quantitative analysis. The RSD value of intraday precision was less 1% for LA, indicating good repeatability of the Q-NMR method.

### Degradation kinetics of LA

**Fig 7:**
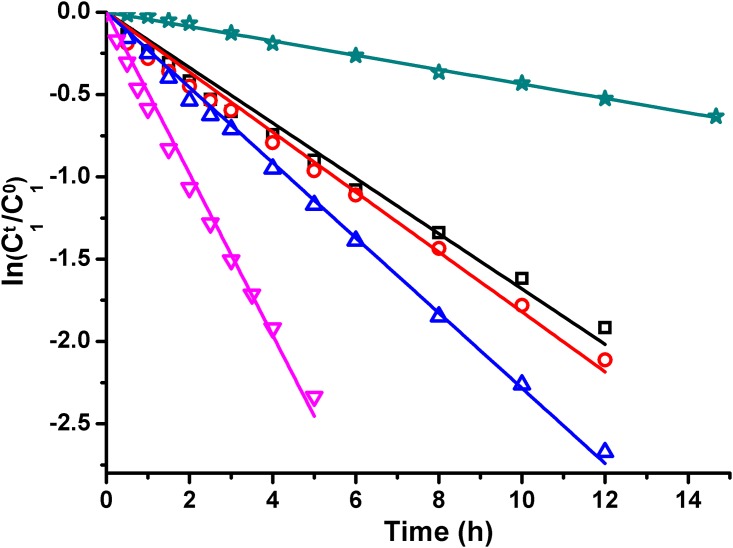
Pesudo-first-order plots for degradation of LA at different experimental conditions. (**☆**)Exp. No. I; (**□**)Exp. No. II; (**▽**)Exp. No.III; (**○**)Exp. No. IV; (**△**)Exp. No. V.

A linear relationship between natural logarithmic remaining percent of LA and degradation time was found as shown in Fig 7. The observed first-order rate constant, *k*_r1_, was determined and the results were shown in Table 2. All the determination coefficients are higher than 0.99, which means that satisfactory fitting was obtained. Degradation constant k_r1_ increased as initial pH value or temperature increased.

The degradation rate constants at different initial pH values were shown in Table 2 (Exp. No. II, IV and V). Initial pH value also showed influences on the degradation rate of LA. LA tended to generate more degradation products as the initial of pH value of phosphate solution increased. Meanwhile stability of Sal A deteriorated with the increasing of solution pH value. These might be the partial factors which caused the variation of degradation rate constants.

The rate constant at various temperatures were also shown in Table 2 (Exp. 1, 2 and 3). Natural logarithmic remaining percent of LA versus degradation time were shown in Fig 7 (Exp. 1, 2 and 3).

The data obtained was fitted with Eq (6) and the results were shown in Table 3. The activation energy and the frequency could be further applied to predict the theoretical half-life of LA solution by assuming a similar mechanism. Half-life of LA at 25°C was predicted to be 2742 days in a phosphate buffer solution (pH 4.75). The prediction rate constant at 25°C was also shown in Table 3.

**Table 3:** Fitting results of Arrhenius equation.

	pH	C (mg/mL)	*E*_a_ (kJ/mol)	ln *A*	*k* (25°C) (h^-1^)	*r*^2^
*k*_r1_	4.75	2.00	132.4±0.9	41.98±0.31	1.053 × 10^−5^	0.9999
*k*_r2_	4.75	2.00	146.1±2.4	46.14±0.81	2.695 × 10^−6^	0.9994

### Degradation kinetics of Sal A

**Fig 8:**
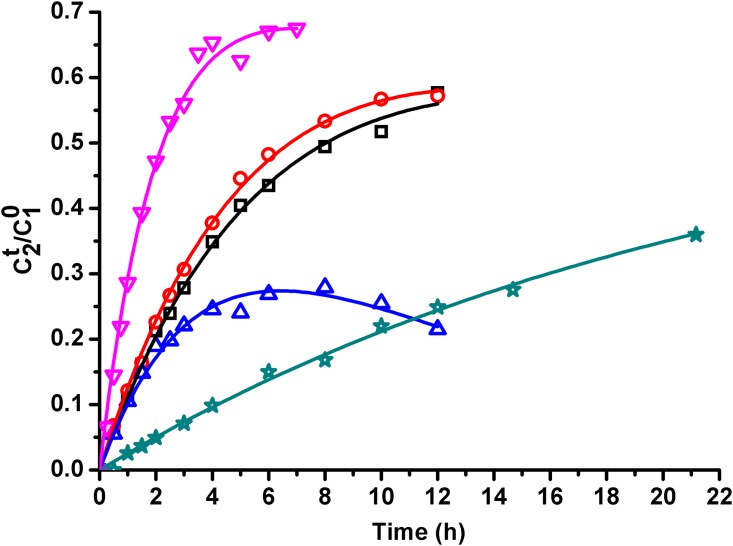
Concentration-time plots of Sal A at various experimental conditions. (**☆**)Exp. No. I; (**□**)Exp. No. II; (**▽**)Exp. No.III; (**○**)Exp. No. IV; (**△**)Exp. No. V.

The time concentration data of Sal A was fitted with Eq (5) and the results were shown in Table 4 and Fig 8. All of the determination coefficients are higher than 0.98, which means that satisfactory fitting was obtained. The results of *k*_r2_/*k*_r1_ were higher than 0.5, indicating over half amount of LA converted to Sal A under our experimental conditions.

**Table 4:** Rate constants of Sal A at various experimental conditions.

Exp. No.	*k*_r2_ (h^-1^)	*k*_r3_ (h^-1^)	*r*^2^	*k*_r2_/ *k*_r1_
I	0.0262±0.0003	0 ^a^	0.997	0.599
II	0.122±0.002	0.0151±0.0034	0.997	0.726
III	0.376±0.009	0.0182±0.0073	0.994	0.766
IV	0.139±0.002	0.0201±0.0026	0.998	0.766
V	0.119±0.003	0.100±0.007	0.984	0.521

^a^ k_r3_ in Exp. I was set to 0 because it was very small.

The degradation rate constants at different initial pH values were shown in Table 4 (Exp. 2, 4 and 5). The results showed the stability of Sal A was highly dependent on initial pH value of phosphate buffer. Sal A easily degradated to other compound in Exp. 5.

The degradation rate constants at different research temperatures were shown in Table 4 (Exp. 1, 2 and 3). The prediction rate constant (k_r2_) at 25°C was shown in Table 3.

## Conclusion

A novel Q-NMR combined with HPLC-MS procedure has been proposed for investigating the degradation process of TCM components using LA solution as a sample system. The proposed method allowed in-situ monitoring of kinetic samples in low oxygen conditions, which was critical for the stability of easily oxidized TCM components, such as Sal A. This NMR method is simple, rapid, specific and no specific reference standards are needed for quantification of target compounds. Therefore this method can be used to investigate TCM systems containing components that are unstable or difficult to prepare.

Eight main degradation products of LA were detected, seven of which were characterized by LC-MS and compared with available reference compounds. These degradation products are proposed to form main pathway of LA, including decarboxylation, ring-open of benzofuran and the hydrolysis of the ester bond in the parent compound.

Kinetic parameters for both LA and Sal A degradation were fitted. The degradation of LA obeyed a pseudo-first-order kinetics. Degradation rate constants increased with increasing of initial pH value or temperatures. Rate constants k_r1_ and k_r2_ obeyed Arrhenius equation. The kinetics studies also indicated that Sal A was the primary degradation product of LA, which was in accordance with the results of Wang et al.’s work [25]. Q-NMR combined with HPLC-MS method will be one of the most promising techniques for investigating degradation process of active components in TCM.

## Abbreviations

Q-NMR: quantitative ^1^H NMR
HPLC-MS: high performance liquid chromatography coupled with mass spectrometry
ESI: electrospray ionization
TCM: traditional Chinese medicine
LA: Lithospermic acid
Sal A: Salvianolic acid A
Sal B: Salvianolic acid B
Sal C: Salvianolic acid C
iso-Sal C: iso-Salvianolic acid C
Sal T/U: mixture of Salvianolic acid T and U
TSP: trimethylsilyl propionic acid
MCR: multivariate curve resolution
